# Minimally Invasive Implantation of an Extravascular Implantable Cardioverter-Defibrillator Device in a 2 Year Old

**DOI:** 10.1016/j.atssr.2024.12.001

**Published:** 2024-12-16

**Authors:** Daniel A. Rabin, Robert M. Huibonhoa, Mark D. Plunkett, Sunita J. Ferns, Harma K. Turbendian

**Affiliations:** 1 University of Illinois College of Medicine, Peoria, Illinois; 2 Pediatric Critical Care, Department of Pediatrics, University of Illinois College of Medicine, Peoria, Illinois; 3 Pediatric and Congenital Cardiac Surgery, Department of Surgery, University of Illinois College of Medicine, Peoria, Illinois; 4 Pediatric and Congenital Cardiac Surgery, OSF Children's Hospital of Illinois, Peoria, Illinois; 5 Pediatric Cardiology, Department of Pediatrics, University of Illinois College of Medicine, Peoria, Illinois

## Abstract

Automated implantable cardioverter defibrillator device implantation in young children is a rare procedure that typically necessitates intrapericardial lead placement. The extravascular implantable cardioverter defibrillator device can be implanted with the patient in a substernal position and with a straightforward and minimally invasive technique. The largest incision is made to accommodate the generator device. The alignment of the coils and device provides a good vector for defibrillation while permitting somatic growth. Here we describe implantation of an extravascular implantable cardioverter defibrillator in a 2-year-old child, among the youngest patients to receive this device to date.

Automated implantable cardioverter defibrillators (AICDs) are implanted in pediatric patients to prevent sudden cardiac death, often the result of congenital heart disease, cardiomyopathies, or inherited arrhythmia syndromes. Very few children receive an AICD for secondary prevention. Of the 500 to 600 pediatric device implantations performed yearly in the United States, only 20% are for secondary prevention.^1^, ^2^, ^3^ In younger patients, in whom transvenous systems are undesirable because of a high rate of complications associated with smaller vasculature, intrapericardial coils have been placed through a median sternotomy, thoracotomy, or subxiphoid approach.^4^ The extravascular implantable cardioverter defibrillator (EV-ICD) device, which was approved for use in the adult population in October 2023, permits minimally invasive placement of a substernal lead. Here we describe the placement of an EV-ICD in one of the youngest patients to date, a 2-year-old boy with a witnessed, out-of-hospital ventricular fibrillatory arrest.

A 2-year-old boy with no previous medical history was successfully resuscitated with no neurologic deficits after a ventricular fibrillatory arrest at home. Whole gene exome testing showed an *SCN5A* mutation associated with Brugada syndrome. On the basis of these events, the patient met a Class 1A indication for AICD placement for secondary prevention.

With the patient in a modified semilateral decubitus position, a 5-cm vertical incision was made in the left anterior axillary line starting at the level of the nipple (Figure 1A). A pocket for the device was created deep to the latissimus dorsi muscle. A 2-cm transverse incision was made at the leftward aspect of the inferior tip of the xiphoid process (Figure 1B). The anterior rectus sheath was incised, and dissection was continued superiorly under the left hemisternum. The manufacturer’s tunneling device was introduced through the incision and advanced superiorly under the sternum by using fluoroscopic guidance. Once in an appropriate position, the tunneling device was removed, leaving the introducer sheath in place. The AICD coil was introduced into the sheath, it was moved into position with the distal tip near the sternal notch and the device resting along the left hemisternum, and the sheath was then removed.

**Figure 1:**
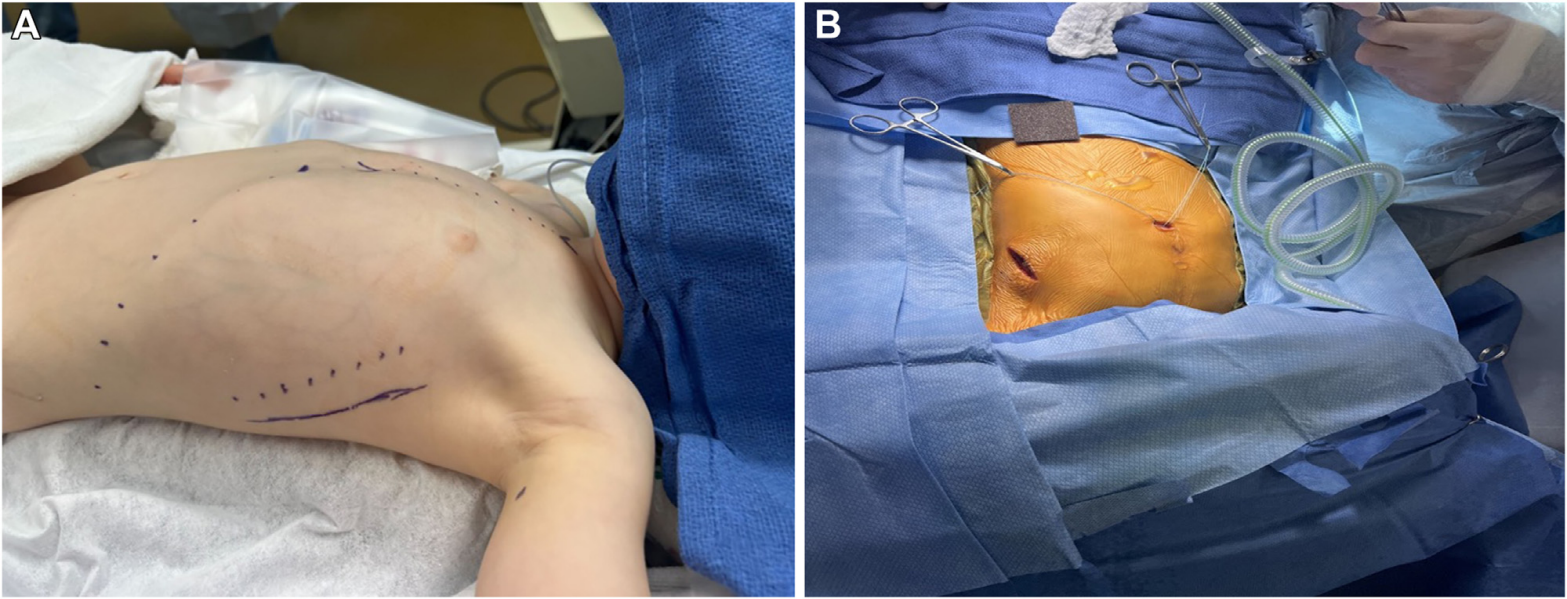
Intraoperative positioning and incisions for implantation of the extravascular implantable cardioverter defibrillator. (A) The procedure was performed with the patient in the modified semilateral decubitus position. (B) The subxiphoid incision was made to introduce the substernal tunneling device. The lateral incision was made for the generator pocket.

The suture sleeve around the lead was secured to the rectus fascia, and the tunneling device was used to guide the lead along the left inferior costal margin and then up the anterior axillary line to the device. This maneuver necessitated a small counterincision at the junction of the left inferior costal margin and the left anterior axillary line. The lead was connected to the device, placed into the pocket, and then secured to the external oblique fascia. Adequate placement and function of the device were confirmed, and all incisions were closed (Figure 2). The patient had an uncomplicated postoperative course and was discharged home the day after device placement. He was readmitted a week later with recurrent ventricular tachycardia (VT) and several successful defibrillations. The VT responded well to quinidine, and he was discharged home on the same day.

**Figure 2:**
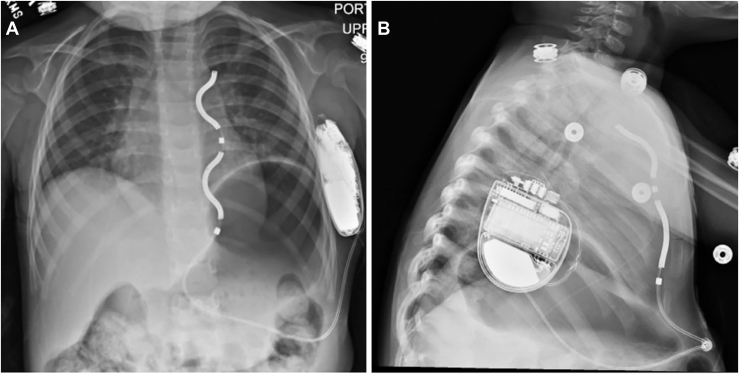
Radiographic confirmation of device placement. (A) Posteroanterior and (B) lateral chest roentgenograms demonstrating appropriate position of the lead and generator.

## Comment

The EV-ICD is a novel device that permits completely extravascular and extrapericardial implantation of a defibrillator device. The technique we describe is straightforward, minimally invasive, and conducive to rapid postoperative recovery, much like earlier reports of epicardial implantable cardioverter defibrillator (ICD) systems.^5^ The largest incision made was to accommodate the size of the generator device. The submuscular pocket below the latissimus dorsi muscle maintained additional superficial tissue coverage over the device in a young child with minimal subcutaneous tissue. The alignment of the coils and device provided an appropriate vector for defibrillation while permitting somatic growth.

The EV-ICD is indicated for adult patients who have limitations or contraindications to standard transvenous ICD (TV-ICD) systems. In the initial pivotal study, the effectiveness in delivering defibrillation therapy at implantation was 98.7%, with no major intraprocedural complications when compared with TV-ICDs and subcutaneous ICD (SC-ICD) systems.^5^ Additional advantages when compared with SC-ICD systems include the ability to deliver antitachycardia pacing (ATP), an important feature that can help avoid defibrillation in patients with stable VT, and a smaller generator with greater battery capacity.

ICDs in younger pediatric patients present several unique challenges, given the age, growth potential, and specific physiologic needs of these patients. Placement of TV-ICD systems in infants and young children is limited by the size of the vasculature and an increased risk of complications. Standard epicardial ICD leads, coils, or plates must be implanted within the pericardium. The size of the generator often limits placement to an abdominal pocket. Epicardial leads traversing 2 body cavities are subject to an increased risk of lead fracture or displacement over time with somatic growth. Traditional epicardial ICD systems are also limited by a temporal increase in defibrillation thresholds, as well as by device failure that can result from alteration in the defibrillation vectors.

Our report describes the implantation of an EV-ICD in one of the youngest patients to date. This system offers several unique advantages for secondary prevention in infants and children. With the entire system in the chest, we hope to mitigate some of the limitations of epicardial systems such as lead failure associated with somatic growth. The complete extrapericardial location facilitates simpler device explantation in cases of device failure, lead fracture, or infection. The vasculature is preserved for potential future implantation of a TV-ICD. The unique 2-coil and ring feature with the EV-ICD lead allows for variable programming options that are beneficial as the child grows. Limitations associated with the EV-ICD include the lack of long-term backup pacing, an important feature in TV-ICD and epicardial ICD systems in patients needing bradycardia therapies. The substernal placement of the lead precludes use of the EV-ICD in patients who have had a previous sternotomy; therefore, this device is not an option in a significant portion of children with congenital heart disease.

In the younger pediatric population with inherent limited options for implantable defibrillators, the EV-ICD offers a safe, easy, and effective alternative option. Long-term follow-up on our patient will determine future efficacy and complications associated with this system with time. Future prospective trials will need to be undertaken to evaluate its ultimate safety and efficacy.
